# Understanding Calabar swellings: Assessing subcutaneous loiasis using ultrasound

**DOI:** 10.1371/journal.pntd.0014240

**Published:** 2026-04-21

**Authors:** Luzia Veletzky, Jeremy T. Campillo, Miveck Pakat, Jessica Kirchner, Irina Gessl, Veronika Vetchy, Jakob Kittinger, Lene Terslev, Richard J. Wakefield, Georgios Filippou, Peter V. Balint, Edoardo Cipolletta, Emilio Filippucci, Malte Jäschke, Giorgio Tamborrini, Markus Herbert Lerchbaumer, Zunaid Karim, Maria-Antonietta D’Agostino, Sebastien Pion, Hannes Platzgummer, Michel Boussinesq, François Missamou, Peter Mandl, Cedric Chesnais

**Affiliations:** 1 Department of Medicine I, Division of Infectious Diseases and Tropical Medicine, Medical University of Vienna, Vienna, Austria; 2 TransVIHMI, INSERM Unité 1175, Institut de Recherche pour le Développement (IRD), Université de Montpellier, Montpellier, France; 3 Programme National de Lutte contre l’Onchocercose, Direction de l’Épidémiologie et de la Lutte contre la Maladie, Ministère de la Santé et de la Population, Brazzaville, Republic of Congo; 4 Department of Internal Medicine III, Division of Rheumatology, Medical University of Vienna, Vienna, Austria; 5 Department of Biomedical Imaging and Image-guided Therapy, Medical University of Vienna, Vienna, Austria; 6 Center for Rheumatology and Spine Diseases, Rigshospitalet, Copenhagen, Denmark; 7 Department for Clinical Medicine, University of Copehagen, Copenhagen, Denmark; 8 Leeds Institute of Rheumatic and Musculoskeletal Medicine, Leeds Biomedical Research Centre and Leeds Teaching Hospitals Trust, Leeds, United Kingdom; 9 Rheumatology Department, IRCCS Galeazzi, Sant’Ambrogio Hospital, Milan, Italy; 10 Department of Biomedical ad Clinical sciences, University of Milan, Milan, Italy; 11 Department of Rheumatology and Clinical Immunology, Semmelweis University, Budapest, Hungary; 12 Musculoskeletal Radiology Group, Medical Imaging Centre, Semmelweis University, Budapest, Hungary; 13 Department of Internal Medicine, Marche University Hospital, Ancona, Italy; 14 Academic Rheumatology, University of Nottingham, Nottingham, United Kingdom; 15 Rheumatology Unit, Department of Clinical and Molecular Sciences, Polytechnic University of Marche, Jesi (Ancona), Italy; 16 Department of Diagnostic and Interventional Radiology, Paediatric Radiology and Neuroradiology, University Medical Center Rostock, Rostock, Germany; 17 UZR - Swiss Ultrasound Center and Institute of Rheumatology, Basel, Switzerland; 18 Department of Radiology, Charité-Universitätsmedizin Berlin, Corporate Member of Freie Universität Berlin and Humboldt Universität zu Berlin, Berlin, Germany; 19 Department of Rheumatology, Mid Yorkshire NHS Trust, Yorkshire, United Kingdom; 20 Catholic University of the Sacred Heart, Rome, Italy; 21 Rheumatology Division, Fondazione Policlinico Universitario A Gemelli, IRCSS, Rome, Italy; Bayer AG, SWITZERLAND

## Abstract

Loiasis is known for two main clinical manifestations: eyeworm and Calabar swellings. Calabar swellings are non-pitting edemas, often on the upper limbs or presenting as unilateral orbital swelling. They are described as itchy or painful, limit mobility, and cause notable morbidity. Although adult *Loa loa* filariae (macrofilariae) are known to migrate widely through host tissues, their exact pathways and tissue preference (tropism) remain unclear. Whilst the exact mechanisms remain unclear, Calabar swellings are considered to be localized allergic reactions to worm antigens, resulting in angioedema. Leveraging advances in portable ultrasound, this project aimed to characterize Calabar swellings via ultrasound to better understand the pathophysiology behind them. This study was conducted in the Lékoumou department, Republic of Congo, as an ancillary investigation within the MorLo (Morbidity due to Loiasis) project. During a follow-up visit in September 2023, participants and interested villagers were invited to present if they had Calabar swellings. Participants completed a standardized questionnaire and were examined by a physician who also performed ultrasound imaging. Twenty-two individuals presented with visible swellings; 8 (36.4%) were classified as “not typical”, 12 (54.5%) as “typical” Calabar swellings and fourteen (63.6%) had subcutaneous nodules on the forearms or wrists, with 8 (36.4%) having both simultaneous swellings and nodules. Ultrasound videos of typical swellings showed nonspecific angioedema, whereas videos of nodules revealed subcutaneous trans-fascial, possibly fluid filled, retentions extending through fascia and crossing anatomical boundaries with mixed-echogenic content. Within these retentions, hyperechoic thread-like structures were visible, displaying autonomous movement in two videos. Their measured diameters corresponded to *L. loa* macrofilariae. The ultrasound findings are most consistent with trans-fascial migration of adult *L. loa* filaria, associated with fascial lesions and fluid retention. The classical Calabar swelling appears closely related to—but not directly over—the underlying lesions. Migration of adult *L. loa* filaria causes a destructive and disabling process that warrants research and public health responses to ensure appropriate care for affected individuals.

## Author summary

Loiasis, also known as “African eyeworm,” is caused by the parasite *Loa loa*. The infection is often associated with Calabar swellings—painful and/or itchy swellings that appear without evident reason, most often on the arms or periorbital on the face. These swellings can limit movement, cause significant morbidity, and are considered to be due to localized allergic reactions to worm antigens. In this study, we used a portable ultrasound device to assess these swellings in patients residing in rural villages of the Republic of Congo. We found that the swellings considered typical for loiasis showed unspecific signs of edema. However, many participants additionally had subcutaneous nodules consisting of mixed-echogenic lesions with hyperechoic thread-like structures inside. These thread-like structures matched the size and appearance of adult *Loa loa* filaria, and in some cases we were able to capture autonomous movement of these adult worms. These findings suggest that adult worms may migrate through tissue in ways that damage the fascia and that this process may trigger the painful swellings patients experience. Understanding this migration behavior of adult worms helps explain the disabling symptoms of loiasis and highlights the need for better treatment and support for affected communities.

## Introduction

The parasitic disease loiasis, caused by infection with *Loa loa*, is receiving more and more attention from researchers and public health stakeholders alike [1–3]. Traditionally the infection is known for its two main symptoms: eyeworm and Calabar swellings (CS). The former is the migration of an adult (macro) filaria under the conjunctiva, causing irritation, pain and visual disturbances. The latter are localized, non-pitting edemas typically occurring on the upper limbs, particularly on the back of the hands and the lower half of the forearms. Although less common, CS may also appear on the face, presenting as unilateral orbital swelling [4]. Regardless of its location, the edema is sharply demarcated and can reoccur several times per month [5]. In literature, a median duration of the edema of 3–5 days is described, though longer episodes may occur. CS may be itchy, painful, or both at the same time [3,5,6]. While erythema has occasionally been described, neither wounds nor puncture sites suggesting an infection site, nor lymphangitis or accompanying lymphadenopathy are present. Importantly, CS affects the mobility of the wrist and metacarpophalangeal joints, leading to functional limitations and significant disease-associated morbidity [3,7]. CS is considered to be localized allergic reactions to worm antigens, resulting in episodes of angioedema [4,5,8,9]. Adult *L. loa* filaria are quite motile and migrate extensively through the host’s tissue. In post-mortem dissections high numbers of adults have been found between the fascia of the lower arms, however the reason for the preference to the upper extremities (tropism) is unclear [10]. Interestingly, the genome of *L. loa* encodes more chemoreceptors compared to other filarial parasites infecting humans, likely facilitating its migrations [11]. Yet, their precise migration pathways, locations of adults during CS episodes, and tissue tropism remain unclear. Recent advances in portable ultrasound devices have expanded their use to resource-limited settings without continuous electricity [12–14]. Building on these advances, this project aimed to assess CS by ultrasound to better understand their pathophysiology and underlying mechanisms.

## Methods

### Ethics statement

The study received approval from the Ethics Committee of the Congolese Foundation for Medical Research (N° 036/CIE/FCRM/2022) and the Congolese Ministry of Health and Population (N° 376/MSP/CAB/UCPP-21). Participants provided written consent before participation in the MorLo study. Individuals not participating in the MorLo study, provided oral consent for ultrasound and questionnaire.

This study was conducted as an ancillary investigation to the MorLo project (Morbidity due to Loiasis), a 3-year (2022–2025) cohort study of 990 individuals in the Lékoumou department, Republic of Congo, designed to evaluate the morbidity associated with loiasis. Study procedures have been described in detail elsewhere [6]. The study region is endemic for loiasis with 20% microfilaremia prevalence, while other filarial pathogens such as *Onchocerca volvulus*, *Wuchereria bancrofti* and *Mansonella* spp. are of low endemicity [15]. The majority of the population work as farmers [7]. During the first annual follow-up visit in September 2023, all participants and interested villagers were invited to present themselves to the study team if they had a CS of the hands or face. Individuals were first assessed by trained field workers for typical symptoms. In case a suspected CS was identified, patients were further examined by a physician. Patients were queried a standardized questionnaire, which included questions on history of eyeworm, symptoms accompanying the swellings, their frequency and duration. The questionnaire is provided in S1 Fig. Based on clinical examination, swellings were categorized into “typical CS” defined as non-pitting transient swellings at the upper extremities or of the orbit, and swellings “not typical for CS”, comprising all other cases. Color photographs were taken of the respective swellings.

### Laboratory examination

Microfilaremia was assessed as previously described [6]. Briefly, 50 µL of daytime blood was collected by finger prick. Thick blood smears (TBS) were prepared, stained with Giemsa, and examined by two experienced technicians to identify *L. loa* microfilaria and quantify the densities..

### Ultrasound examination

Ultrasound examinations were performed using a handheld ultrasound device (Butterfly IQ 2018, Butterfly Network, USA) connected to a smartphone. Grey-scale settings were optimized for superficial structures with a frequency range of 1–10MHz. Longitudinal and transverse scans of the volar aspect of the wrist and distal forearm were performed, and grey-scale videos were acquired using the clinical swelling as a landmark. Grey-scale videos (cineloops) were stored for later analysis. Doppler imaging was not performed due to technical issues. Video duration ranged from 20 seconds to two minutes. The ultrasound scans were performed by LV (infectious disease resident) after prior training by PM (rheumatologist expert in musculoskeletal ultrasound) and HP (musculoskeletal radiologist), both with long-standing expertise in musculoskeletal ultrasound. All ultrasound videos were first reviewed by LV and PM after completion of the study. Videos with pathological findings were further reviewed by HP, who was blinded to the previous expert assessment. Written interpretations from both ultrasound specialists were merged by LV, and final consensus versions were established.

### Reliability study

A small-scale reliability study was performed using a dataset containing 8 grey-scale videos from different individuals from the study. Of those, 4 videos contained pathological lesions with moving structures as identified by consensus of PM and HP, while 4 were deemed as not containing such lesions, this was used as the comparator/gold standard for the reliability study. Experts in musculoskeletal ultrasound (rheumatologists and radiologists) were invited to join this study and to independently review the videos in two rounds, a minimum of two weeks apart, blinded to each other’s and their own previous assessments. The videos were numbered, randomly ordered, and reordered for the second round. For each video, the experts had to answer yes/no to the following question: “In this ultrasound video acquired from the volar aspect of the forearm, can you confirm the presence of a mixed echogenicity lesion with autonomous movement, resembling a fluid-filled cavity with a moving worm-like structure inside?”. Results were first analyzed by descriptive statistics.

Next, intra-rater and inter-rater reliability were calculated using Fleiss’ and Cohen’s kappa. Finally, the percentage of observed agreement (i.e., percentage of observations that obtained the same score [yes or no]) was calculated. All calculations were performed using SPSS (IBM, Version 29.0.2.0). Kappa coefficients, which can range from -1 to +1, were interpreted as follows: < 0 indicate no agreement, and 0-0.20 represent slight; 0.21-0.40 fair; 0.41-0.60 moderate; 0.61-0.80 good and >0.80 excellent reliability [16].

## Results

### Study population/overview

**Fig 1:**
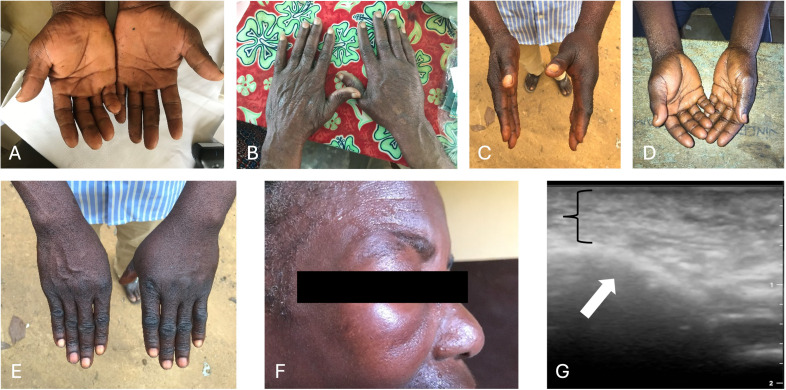
“Typical” Calabar swellings seen during the study of hands (A-E) and face (F) as well as an ultrasound image (G: white arrow indicating the bone, black bracket indicating the edema) of the orbital swelling depicted in (F). Pictures A to F were taken by LV.

Overall, 22 individuals had visible swellings identified by the fieldworker and were further examined clinically and by ultrasound. Details on the study population are shown in Table 1, and Fig 1 provides pictures of “typical CS” observed during the study. A more comprehensive overview, including detailed descriptions of the swellings is provided in S1 Table.

**Table 1:** Overview on the study population including baseline demographics, reported history of eyeworm and of CS, *L. loa* microfilarial densities, presence of typical CS, presence of other swellings not typical for CS, and appearance of nodules. Notably, two individuals (8 and 16) presented themselves as having swellings but were assessed as having only nodules by the clinician. * typical CS defined as non-pitting transient swellings on the upper extremities or near the orbit, ** other swellings defined as all not consistent with the morphology of the above-mentioned description, *** CS near the orbita. M = male, F = female, MD = missing data.

Case Number	Sex	Age (years)	Lifelong eyeworm history	CS history N° lifetime	Microfilarial density (mf/mL)	Typical CS*	Not typical for CS**	Subcutaneous nodules
**1**	F	40	MD	MD	MD	No	Yes	Yes
**2**	F	46	Yes	12	0	No	Yes	Yes
**3**	F	55	Yes	8	0	Yes	No	Yes
**4**	F	47	Yes	10	20	Yes	No	Yes
**5**	M	62	Yes	10	27 640	Yes	No	Yes
**6**	F	53	Yes	0	260	No	Yes	Yes
**7**	F	60	Yes	10	22 320	No	Yes	Yes
**8**	F	72	Yes	0	0	No	No	Yes
**9**	F	63	Yes	20	0	Yes	No	Yes
**10**	F	50	Yes	MD	MD	Yes	No	No
**11**	F	50	MD	MD	0	Yes	No	Yes
**12**	M	85	Yes	10	520	Yes	No	No
**13**	F	59	Yes	1	28 100	Yes***	No	No
**14**	F	31	No	0	3 800	No	Yes	No
**15**	F	68	No	0	6 540	No	Yes	No
**16**	F	43	Yes	MD	MD	No	No	Yes
**17**	F	35	Yes	MD	MD	Yes	No	Yes
**18**	M	44	No	4	0	Yes	No	No
**19**	M	54	No	0	5 760	Yes	No	No
**20**	M	69	Yes	2	0	Yes***	No	No
**21**	F	40	Yes	MD	MD	No	Yes	Yes
**22**	F	46	No	0	0	No	Yes	Yes

Based on clinical examination and provided history of the swelling, 8 of the 22 individuals (36.4%) had swellings that were classified as other swellings “not typical for CS” and 12 (54.5%) were classified as “typical CS”. Of those, two individuals had a facial CS presenting as unilateral orbital swellings (0.9%). Additionally to the “typical CS”, 14 (63.6%) individuals had subcutaneous nodules on the forearms and wrists. Simultaneous presence of CS and subcutaneous nodules was seen in 8 individuals (36.4%). Two individuals had only subcutaneous nodules (0.9%), which they referred to themselves as swellings. These nodules presented as bulging elastic lesions of several cm in length and about one cm in diameter and an elevation of some millimeters above the surrounding skin. Clinically, the “nodules” were reported as painful, beginning with an itchy sensation, appearing repeatedly at similar locations, affecting the same extremity, and disappearing after a few days. In all cases the dominant hand was affected.

### Clinical and ultrasound results

A total of 56 ultrasound videos from 22 individuals were assessed for relevant findings and pathologies. During this review, it became evident that the ultrasound videos of the “typical CS” showed non-specific subcutaneous and muscular edema, with no further remarkable findings, such as foreign bodies or effusions. In contrast, the ultrasound videos of the nodules showed more complex findings, namely subcutaneous retentions of varying echogenicity. These videos were assessed by both specialists (PM, HP). Finally, videos from three individuals, namely Patient 5, 16 and 17, were selected for in-depth evaluation as they showed the best quality. Both specialists provided written descriptions and interpretations of these videos as well as an overarching summary, blinded to each other’s results.

**Fig 2:**
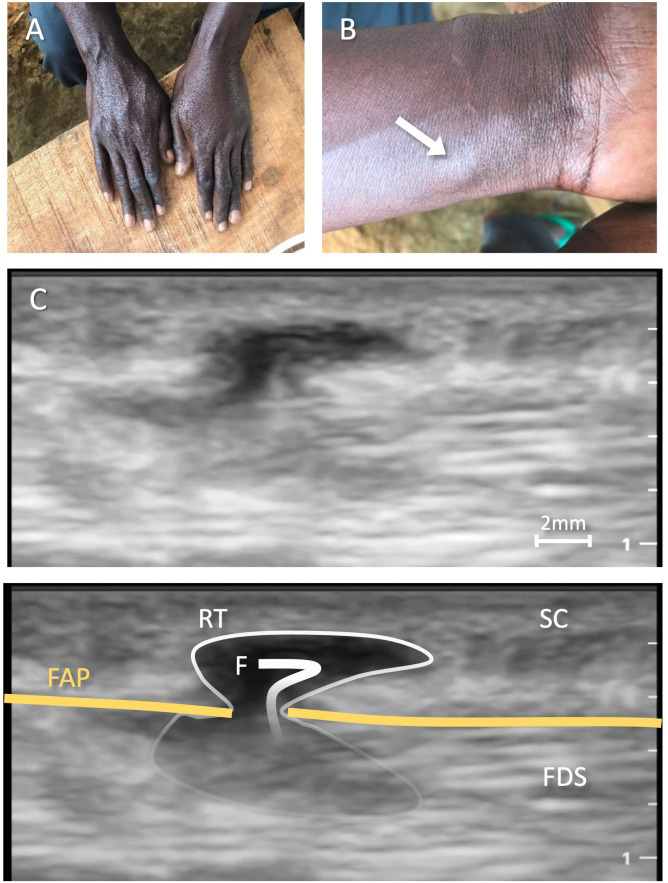
Findings of Patient 5: A) Typical Calabar swelling on the left hand; B) Subcutaneous nodule on the volar aspect of the left distal forearm; C) Corresponding ultrasound image revealing a subcutaneous, anechoic lesion presenting as retention, with trans-fascial gap and hyperechoic structure inside the retention. The thread like structure is less than 0.5 mm in diameter (compare to the 2 mm scale bar, still image from S2 Videos at second 14); **D)** Corresponding ultrasound image with annotations. Legend: RT = retention; F = filaria; SC = subcutaneous tissue; FAP = *palmar antebrachial fascia* (orange); FDS = *flexor digitorum superficialis muscle*. Pictures A and B were taken by LV.

**Fig 3:**
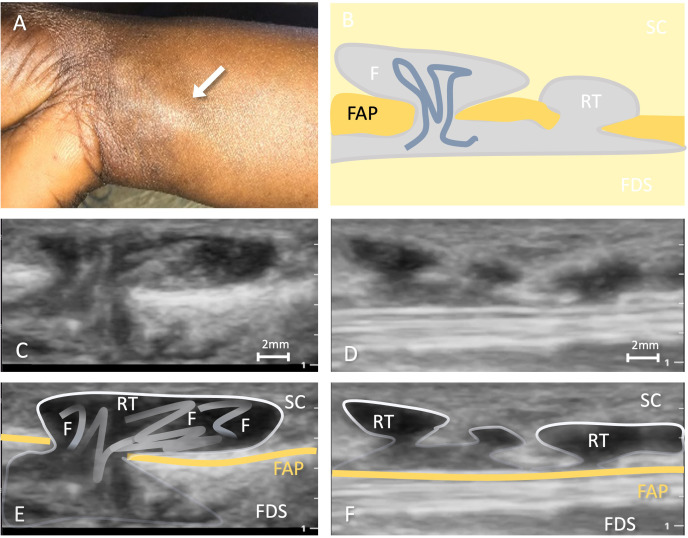
Findings of patient 16: A) Subcutaneous nodule on the right volar forearm indicated by white arrow; B) the corresponding ultrasound image showing a transversal cut of the subcutaneous nodule presenting as an anechoic retention in the ultrasound, with trans-fascial gap and hyperechoic structure inside (still image from S3 Video at second 15) and D) showing another transversal cut of the same nodule depicting multiple trans-fascial gaps (still image from S4 Video at second 6); C and E) Corresponding ultrasound images with annotations; F) a schematic depiction of the findings on ultrasound. Legend: RT = retention; F = filaria; SC = subcutaneous tissue; FAP = *palmar antebrachial fascia* (orange); FDS = *flexor digitorum superficialis muscle*. Picture A was taken by LV.

**Fig 4:**
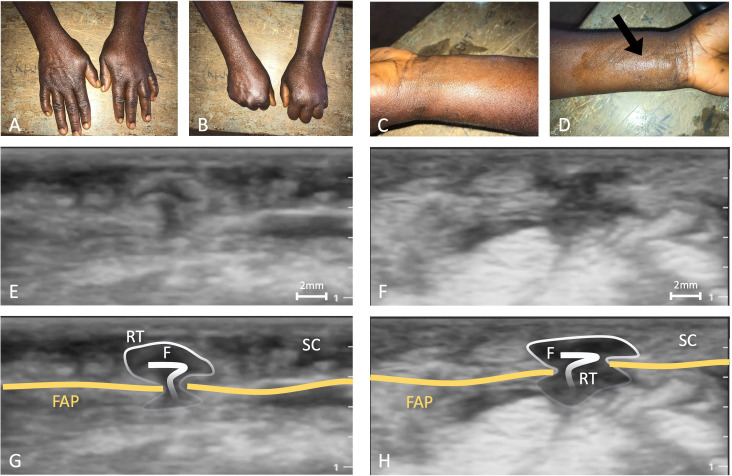
Findings of patient 17: A + B) typical Calabar swelling on the left hand: both hands in an expanded position (A) and with fists closed (B); C + D) subcutaneous nodule on the left palmar forearm, indicated by black arrow (D); E + F) the corresponding ultrasound images showing the subcutaneous nodule presenting as a retention in the ultrasound, with trans-fascial gap and hyperechoic structure inside in longitudinal (E) and transversal scan (F) (both show still images from S5 Video at seconds 3, respectively); and G + H) Corresponding ultrasound images with annotations. Legend: RT = retention; F = filaria; SC = subcutaneous tissue; FAP = *palmar antebrachial fascia* (orange); FDS = *flexor digitorum superficialis muscle*. Pictures A to D were taken by LV.

Summarizing, the experts agreed that these videos showed subcutaneous retentions with structures of varying echogenicity inside. The retentions are transfascial, in that they penetrate the superficial fascia representing holes in this anatomic structure (see Figs 2–4). The expansion of the retentions through the fascia is disregarding the anatomical boundaries and is characteristic of pathological rather than physiological structures (see still images captured from videos in Figs 2C, 2D, 3C–3F and 4E–4H). In one video, several transfascial gaps are visible (S3 Video, see Fig 3D). Within the retentions, hyperechoic structures are visible, which have a thread-like shape. The hyperechoic structures are clearly visible inside the otherwise anechoic retentions. In two videos, from Patient 5 (S2 Video) and Patient 17 (S5 Video) respectively, autonomous movement of the hyperechoic structures inside the retentions is visible. The measured diameters correspond in size to *L. loa* macrofilariae, whose length is typically 22–36 mm (male) and 38–65 mm (female), and whose maximum diameter is 0.4-0.5 mm [17]. Of the three individuals, two had simultaneous “typical CS” (5 and 17) and the third (16) reported that the CS had just eased the day before. For details on ultrasound descriptions and clinical complaints, see individual patient descriptions below. Plain and edited videos are provided in the S1–S5 Videos files, edited versions include a time display, circles indicating lesions and asterisks when movement can be seen.

### Individual patient descriptions

#### Patient 5.

This patient reports a CS about once per month, which stays for 4–5 days and is itchy. The CS always appears at the left arm along with the subcutaneous nodules and he describes a feeling that a worm moves inside, especially after scratching. There is no obvious reasons in his opinion that explains the CS and nodules.

**S2 Video:** This video shows a longitudinal image of the volar aspect of the left distal forearm. Subcutaneous tissues, bones and fascia are visible. As the transducer is moved, an anechoic trans-fascial gap and accompanying anechoic retention becomes visible at a depth of 0.2-0.4 cm during seconds 1–5 and 8–20. Inside the retention is a thread-like, hyperechoic structure that moves from the superficial subcutaneous structures through the fascial gap from seconds 8–16. There is no compression effect visible that would have passively displaced the tissue or caused the movement. The structure diameter is at maximum 0.5 mm, with a length of at least 6 mm, possibly longer (see Fig 2C and S2 Video). Differentials would be a blood clot in a superficial vein; however, this is not a suitable explanation as in this case no autonomous movement would occur.

#### Patient 16.

This patient reported extreme pain associated with the nodule, spreading to the fingers affecting the region innervated by the median nerve. She reported a swelling consistent with the morphology of the description of a “typical CS” on the same hand that had eased the day before.

**S3 Video:** This video shows a longitudinal image of the volar aspect of the right distal forearm. There is a subcutaneous retention of mixed-echogenicity transversally cut with a transfascial gap over a total length of about 1 cm at a depth of 0.2-0.6 cm, appearing in the video at seconds 0–8, 13–35 and 41–51. The fascial gap appears as pinhole, with a fascial penetration up to 0.4 cm in diameter. The fascial gap does not respect anatomical boundaries, giving the impression of a break through the superficial fascia. There are transversally oriented structures in the fascia gap and longitudinally oriented structures in the retention. At seconds 0–3 movements can be recognized, however these could also be artefactual, i.e., passively caused by pressure applied by the transducer. Later in the video there are movement artefacts thus autonomous movements are not clearly seen.

**S4 Video:** This video shows a longitudinal image of the volar aspect of the subcutaneous retention located at the left distal forearm. There are subcutaneous retentions of mixed-echogenicity transversally cut with multiple trans-fascial gaps over a total length of 1.5 cm at a depth of 0.2-0.6 cm. At seconds 3–4 trans-fascial movement can be detected; however, this movement could be passive, i.e., due to pressure applied by the transducer. At second 7 a large subcutaneous retention becomes visible with hyperechoic thread-like structures inside. Between seconds 13–19 there are possible movements within the retention. At seconds 25–30 the retention and wormlike structure becomes visible again but without movement. The thread-like structure is diagonally cut, seems to be 0.5 mm thick and is localized within the retention. The length of the structure cannot be measured as it is never seen in its complete length.

#### Patient 17.

The patient reports a CS that has repeatedly affected the left hand, it has been there for nearly two months now. The CS is itchy and she experiences cramps. The swelling hinders her to completely close the fist (also depicted in Fig 4B). The nodules are very painful and occur simultaneously as the CS.

**S5 Video:** This video shows longitudinal images of the volar aspect of the lower left arm. The antebrachial fascia is affected and visible in a longitudinal section. There is a retention with a mushroom-like shape at seconds 3–4. At seconds 10–12 a pipe shaped lesion becomes visible and at second 11 movement can be detected; but this could be due to transducer pressure and an artefact. However, at the same site at seconds 13–19 the lesion becomes again visible and at seconds 13–17 there is autonomous movement from the superficial into deeper areas.

### Results of the reliability study

A total of 13 experts participated in the reliability study. The experts had to assess 8 videos in two rounds comprising the four videos presented above and 4 videos from the dataset that did not show clear subcutaneous lesions as agreed consensually (see Methods). This led to an absolute number of 208 assessments, 26 assessments for each video. Overall, 81.7% (170/208) were correctly assessed.

All 13 experts agreed in both rounds that the video of patient 5 (26/26, 100%) showed “the presence of a mixed-echogenicity lesion with autonomous movement, resembling a fluid-filled cavity with a moving worm-like structure inside”. The first and second videos of patient 16 (S3-S4 Video files) were rated in 73.1% (19/26) and 76.9% (20/26) of assessments as showing the finding of interest, respectively. The video from patient 17 (S5 Video) was considered positive in 88.5% (23/26) of assessments. Of all videos without the finding of interest, 21.2% were falsely categorized as positive (22/104), and 95.5% of these false positives concerned two videos (21/22).

Inter-rater reliability calculated by Fleiss’ Kappa of round two was 0.498 (95% CI 0.404 - 0.591) with an observed agreement of 92.95% (range 54.55%-100%). Intra-rater reliability calculated by Cohen’s kappa was 0.795 (95% CI 0.669-0.909; p < 0.001) with an observed agreement of 89.77% (range 62.5%-100%).

## Discussion

This project aimed to assess a typical sign of loiasis, the so-called Calabar swelling (CS), using ultrasound. CS has previously been described as a major factor in loiasis-related morbidity as it occurs frequently and significantly limits hand function [3,6,7]. Today, loiasis is increasingly seen as a disease of relevance and may be recognized as a neglected tropical disease by the World Health Organization in the future [1]. Understanding its pathophysiology is thus becoming increasingly important to develop adequate and effective treatment to truly be able to improve the daily life of affected individuals.

CS have been described early on in loiasis case reports and are named after a Nigerian town, where this sign was first reported in Western literature in 1895 [18]. Traditionally, they are described as “transient, non-pitting edema occurring at hands and wrists” and are considered to occur as a localized allergic reaction to the parasite antigen [8,9]. However, the exact mechanism, and why CS tends to occur above the wrists and hands have never been fully understood. In endemic regions CS are often treated by homemade or traditional remedies, including hot wrappings, hot water or creams as well as anti-inflammatory drugs [19]. Currently there are no specific treatment guidelines available for CS. A better understanding of the mechanisms associated with CS pathology may help develop and evaluate new specific treatment strategies.

Advances in ultrasound technology allow higher resolution of soft tissue and have been used to visualize macrofilariae of various species. Live and moving macrofilariae of *Wuchereria bancrofti* and *Brugia* species can be visualized in the lymphatic system by ultrasound - a finding also known as the filarial dance sign [20,21]. Several case reports on ultrasound detection of subcutaneously residing *Dirofilaria* spp. macrofilariae have been published [22,23] and ultrasound of *Onchocerca* nodules also showed moving filaria [24], specifically echogenic filarial structures in fluid-filled retentions. Otherwise, there is only limited literature of *L. loa* macrofilariae residing in subcutaneous tissue; one case report described a pipeline-shaped lesion as an ultrasound finding in the subcutaneous tissue at the calf of a patient with loiasis consistent with the morphology of a macrofilaria [25].

**Fig 5:**
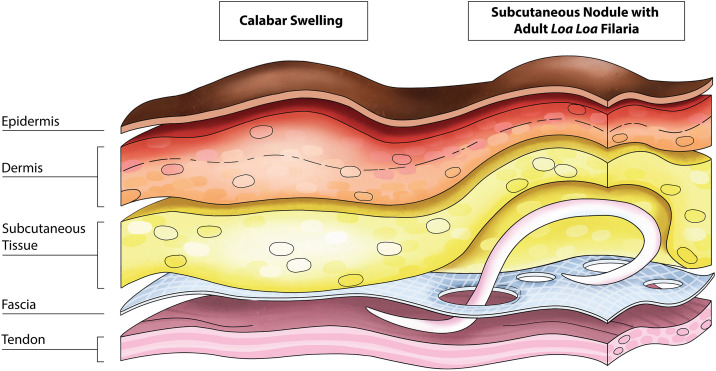
A schematic presentation of the trans-fascial migration of an adult *L. loa* parasite(macrofilaria), including fascial lesions and a fluid retention. The figure is original work created by the authors. The authors are the sole copyright holders and grant permission for publication of the figure under the Creative Commons Attribution 4.0 (CC BY 4.0) license.

Apart from case reports, CS themselves have never been systematically examined by ultrasound. Postmortem autopsies performed in 1905 on a *L. loa*-infected individual showed 34 *L. loa* macrofilariae residing in the fascial layers of the palmar forearms [10]. Here, based on previous descriptions, we hypothesized that macrofilariae may be detected by ultrasound around or next to CS [25–28]. Importantly, during the project, physical examination revealed subcutaneous nodules on the palmar side of the wrist in a relevant proportion of patients suffering from typical CS. The nodules were described as painful, acutely occurring at the volar distal forearm. Patients stated that the occurrence of the nodules coincides with swelling of the dorsal and palmar side of the hand, which is consistent with the classic clinical picture of CS [4]. All affected patients reported that the nodules would occur several times per year. To our knowledge these nodules have not been analyzed by ultrasound or systematically described in the literature before. In ultrasound these nodules presented as anechoic retentions with hyperechoic filarial like structures inside. In two ultrasound videos we were able to detect autonomous movement of filaria-like structures through holes in the fascial layers of the forearm. The ultrasound findings of our study suggest that these structures actively penetrate fascial layers and thus cross anatomical barriers. The structures seem to move from the subcutaneous tissue to deeper layers of tissue, e.g., tendon sheaths, away from the transducer while applying pressure. The structures were not visualized in their entire length, but their estimated size would be 0.5 mm in diameter and a minimum of 6 mm in length, probably longer. This would be consistent with the size of a *L. loa* macrofilaria [17,27]. We therefore hypothesize that *L. loa* macrofilariae cause these described nodules by penetrating the superficial fascia of the forearm, thereby causing pain and functional disability. Fig 5 schematically displays this finding.

This has been further supported by the small-scale reliability study, which found moderate to good reliability among a group of international experts on small-part ultrasound. While the overall reliability was limited, the agreement on the finding in the video from patient 5 (S2 Video) was 100% in both rounds, and false positivity was mainly driven by two videos, which limited the overall outcome of the reliability study.

Trying to understand the pathology behind this finding, it is important to note that the affected region is an anatomical site with special features. The tight situs in the volar wrist region causes a close relation of fasciae, nerves and muscles with hardly any space in between [29]. Thus, a filarial migration might lead to increased pain and functional restriction. Interestingly, all affected individuals reported occurrence of both CS and nodules on their dominant hand. The affected individuals are all residents of rural areas working in subsistence farming. This implies hard physical labor, which puts significant strain daily on their wrists and hands. It seems reasonable that physical stress may additionally force the *L. loa* filariae to move. Another aspect could be that individuals may have chronic tendinopathies, such as recurrent tenosynovitis possibly predisposing these areas for worm migration and facilitating further damage by the parasite. The classical presentation of CS may be a secondary reaction to the process, driven by local inflammation. Further supporting this theory, it has been described that CS can be induced by a knock at the site, or even by rubbing the skin, which may damage the worm, leading to the release of antigens and subsequently causing the angioedema [8,9]. The extensive tissue swelling is, however, not directly above the subcutaneous nodules. This may be explained by the fact that the soft tissue lying superficially directly above the carpal tunnel is extremely thin in contrast to the superficial palmar areas where muscles and subcutaneous tissue are thicker and constitute more space for swelling [29].

Anecdotal reports from affected individuals describe the impact of physical pressure on adult worm (macrofilarial) movement in loiasis, including the prevention of eyeworm migration if pressure is applied, thereby providing a physical barrier and preventing migration of the adult worm into the subconjunctival space (personal communication). Further, in highly endemic areas, patients are encouraged to keep their heads down to keep the worm under the conjunctiva while waiting for surgical removal [5,30]. In our study, the filarial structures tended to move away from the ultrasound transducer from the more superficial tissues towards the deeper layers. Since the ultrasound transducer must be applied to the skin with a certain amount of pressure, this also corresponds to the image of a moving filaria reacting to physical impact.

We did not surgically remove the macrofilariae seen in the ultrasound examination due to limited resources and ethical considerations and thus cannot prove that the documented structures are definitely *L. loa* macrofilariae. However, the affected individuals reside in highly endemic areas for loiasis and, among the three individuals with convincing ultrasound findings, two experienced simultaneous typical CS and nodules, and the third reported a swelling that had just eased. While some experts would consider CS as diagnostic for the infection, we propose to discuss further possible differentials. Other filariae such as *Onchocerca volvulus*, *Wuchereria bancrofti* and *Mansonella* spp. are of low endemicity in the Lékoumou district. The clinical presentation of these macrofilariae is different, as they cause subcutaneous stationary, persistent nodules in onchocerciasis or reside in the lymphatics in the case of lymphatic filariasis, respectively [15,31]. *Mansonella* spp. may be similar in size but are of low endemicity in the study area. *Dirofilaria* spp. have been described to cause subcutaneous nodules similar to the ones described here; however, they are considered to be non- endemic in the study area. Non-parasitological differential diagnoses would be thrombosis or inflammatory phenomena such as gouty tophi, other infections, or destructive processes. However, due to the documented autonomous movements in two different cases and the slender shape of the structures within the retentions, these are very unlikely.

Next to the missing parasitological confirmation of the finding, there are other limitations that warrant discussion. First, for microfilaria detection we used calibrated thick blood smears but no concentration method, which may have led to missing some microfilaremic individuals. Next, we assessed swellings at the upper extremities and face but not on other body parts. This was done for simplicity of the field work and may have missed CS on other body parts. However, this study did not aim to assess the true prevalence of CS but ultrasound findings of the typical clinical presentation. Further, the quality of the ultrasound videos is limited as we used a handheld transducer connected to a smartphone. Thus, during the ultrasound, pictures were only seen on a small screen on the phone, making direct interpretation in the field difficult. Further, we experienced some issues of connection, and some ultrasound videos were stalled several times. Unfortunately, the Doppler function of the handheld ultrasound transducer was not working. Future studies should use better ultrasound machines to allow recording of more detailed videos. Finally, while the design of the reliability study was based on previous work from the Outcome Measures in Rheumatology Ultrasound Working Group [32], the video dataset was limited by the small sample size of the study. Next steps include the development of consensual definitions of the filaria-like lesions on ultrasound, a potential scoring system - binary, semiquantitative, or quantitative - and the replication of the reliability study on a larger video dataset. This would be followed by a reliability exercise on patients, including the acquirement of videos and real-time scoring of lesions [33].

## Conclusion

Considering the ultrasound findings and clinical complaints reported by the patients, we believe that our results are most consistent with a trans-fascial migration of adult *L. loa* parasites, associated with fascial lesions and fluid retentions. The classical clinical picture of Calabar swelling seems to appear in close relation but not directly above these lesions. Loiasis is a complex disease, with typical clinical manifestations caused by migration of adult worms and Calabar swellings are not just benign swellings. They may indicate an underlying destructive and disabling process and therefore need to be addressed by further studies and public health programs to provide adequate treatment to affected individuals.

## Supporting information

S1 Table This Table provides the study data. (DOCX)

S1 Fig This file contains the study questionnaire. (DOCX)

S1 Video This file contains the original, unedited versions of the videos. (TAR)

S2 Video This file is the edited version of the video of patient 5. (MP4)

S3 Video This file is the edited version of the first video of patient 16. (MP4)

S4 Video This file is the edited version of the second video of patient 16. (MP4)

S5 Video This file is the edited version of the video of patient 17. (MP4)
