# Supplementary material for: Understanding Calabar swellings: Assessing subcutaneous loiasis using ultrasound
Source: PLoS Negl Trop Dis. 2026 Apr 21;20(4):e0014240. doi: 10.1371/journal.pntd.0014240 (PMC13124053; doi:10.1371/journal.pntd.0014240)
Supplement: S1 Table — (DOCX) [file pntd.0014240.s001.docx]

**S1 Table**

| **Case Nr** | **Sex** | **Age (years)** | **Eyeworm history** | **CS history (N°)** | **Microfilaremia (mf/mL)** | **Typical CS** | **Other swelling** | **Description of swelling** | **Subcutaneus**  **nodules** | **Description of nodules** |
| --- | --- | --- | --- | --- | --- | --- | --- | --- | --- | --- |
| 1 | F | 40 | MD | MD | MD | No | Yes | Left arm, monthly appearance duration 6 days, itchy and painful | Yes | painful, elastic, fixed, on the distal forearm, size 0.5x0.5cm, duration 6 days |
| 2 | F | 46 | Yes | 12 | 0 | No | Yes | Left hand, monthly appearance duration 2 days, itchy and painful | Yes | painful, elastic, fixed, on the distal forearm, size 0.7x0.7cm, duration 2 days ** |
| 3 | F | 55 | Yes | 8 | 0 | Yes | No | Right hand, bi-monthly appearance duration 2 days, itchy and painful | Yes | painful, elastic, fixed, on the distal forearm, size 0.5x0.5cm |
| 4 | F | 47 | Yes | 10 | 20 | Yes | No | Right hand, itchy and painful, permanently | Yes | painful, elastic, fixed, on the distal forearm, size 2x0.7cm permanently, duration 2 days |
| 5 | M | 62 | Yes | 10 | 27 640 | Yes | No | Left hand, monthly appearance duration 4-5 days, itchy | Yes | itchy, elastic, fixed, on the distal forearm, size 4x1.5cm , duration 4 days |
| 6 | F | 53 | Yes | 0 | 260 | No | Yes | DM | Yes | Right hand, itchy, painful, fluctuant, fixed, on the distal forearm, size 2x1.5cm, duration 6 days |
| 7 | F | 60 | Yes | 10 | 22 320 | No | Yes | Right hand | Yes | itchy, painful, hard, fixed, on the distal forearm, size 2x1.5cm, duration 1 day |
| 8 | F | 72 | Yes | 0 | 0 | No | No |  | Yes | Not itchy, not painful, elastic, on the distal forearm, size 2x1cm, duration permanent for 2 months |
| 9 | F | 63 | Yes | 20 | 0 | Yes | No | right hand, painful | Yes | painful, elastic, fixed, on the distal forearm, size 1x1cm, duration 1 day** |
| 10 | F | 50 | Yes | MD | MD | Yes | No | Right hand, monthly appearance duration 2 days, itchy, painful | No |  |
| 11 | F | 50 | MD | MD | 0 | Yes | No | Right hand, monthly appearance duration 2 weeks, itchy, painful | Yes | Itchy, elastic, fixed, on the distal forearm, size 0.7x0.7cm, duration 2 weeks** |
| 12 | M | 85 | Yes | 10 | 520 | Yes | No | Right hand, itchy, painful | No |  |
| 13 | F | 59 | Yes | 1 | 28 100 | Yes* | No | Right eye, every 2 months appearance duration 6 days, itchy, disturbs vision | No |  |
| 14 | F | 31 | No | 0 | 3 800 | No | Yes | Right hand, monthly appearance duration 7-21 days, itchy, painful | No |  |
| 15 | F | 68 | No | 0 | 6 540 | No | Yes | Both hands, monthly appearance duration 3 days, itchy, | No |  |
| 16 | F | 43 | Yes | MD | MD | No | No | Not at the time of presentation, but eased off the day before. | Yes | On the right distal forearm, several times per month, elastic, fixed,  painful, itchy |
| 17 | F | 35 | Yes | MD | MD | Yes | No | Left hand, appears frequently, itchy | Yes | on the distal forearm, elastic, fixed, extremely painful 2x1cm |
| 18 | M | 44 | No | 4 | 0 | Yes | No | Right hand, every 3 months duration 7-21 days, itchy, painful | No |  |
| 19 | M | 54 | No | 0 | 5 760 | Yes | No | left hand, monthly appearance duration 3 days | No |  |
| 20 | M | 69 | Yes | 2 | 0 | Yes * | No | Left eye, 1 x per year duration 3 days, itchy | No |  |
| 21 | F | 40 | Yes | MD | MD | No | Yes | left hand, monthly appearance duration 3 days, painful, itchy | Yes |  |
| 22 | F | 46 | No | 0 | 0 | No | Yes | multiple swellings of different sizes and body parts | Yes | left distal forearm, painful, 1x2-3cm** |

MD = missing data, * orbital Calabar swelling; MD, missing data, ** appears simultaneously with the Calabar swelling.
