# Supplementary material for: Understanding Calabar swellings: Assessing subcutaneous loiasis using ultrasound
Source: PLoS Negl Trop Dis. 2026 Apr 21;20(4):e0014240. doi: 10.1371/journal.pntd.0014240 (PMC13124053; doi:10.1371/journal.pntd.0014240)
Supplement: S1 Fig — (DOCX) [file pntd.0014240.s002.docx]

**S1 File**

**1. Questionnaire**

**Questionnaire CalSuS**

ID :

Sex :

Age :

1. **Œdème** o Non o Oui
2. Si oui, où ? _____________Quel membre ? ____________
3. Si oui, ça resté pour combien de temps ? ____________
4. Si oui, ça vient combien de fois ? ____________
5. o ça gratte o douloureuse
6. est ce qu’il y avait le vers sur la peu qui sont passés ?
7. Notes :

Documentation : o photo o écho : o vidéo longitudinale

o vidéo transversale o vidéo doppler o autre :_____________

1. **Formation sous-cutanée :** o Non o Oui

Description clinique :

1. Description clinique : o endroit : ___________ o ça gratte o douloureuse o dur o élastique o fluctuant o fixe o mobile o taille : _________cm o fistule o autre :
   1. Ça arrive combien de fois ?________________
   2. Ça reste pour combien de jours ? ________________
   3. Avez-vous eu de fièvre dans le même temps ? o Non o Oui
   4. Il y avait un ganglion correspondant ? o Non o Oui
   5. Piqueur dans le même temps ? o Non o Oui
   6. Un autre chose qui est arrivé dans le même temps ? o Non o Oui

Si oui, décrivez : _________________________________________

Documentation : o photo o écho : o vidéo longitudinale

o vidéo transversale o vidéo doppler o autre :_____________

3. Examen clinique, décrivez les résultats :

Date :
